# Preference Index of Sustainable Natural Fibers in Stone Matrix Asphalt Mixture Using Waste Marble

**DOI:** 10.3390/ma15082729

**Published:** 2022-04-07

**Authors:** Sandeep Singh, Mohammad Iqbal Khairandish, Mustafa Musleh Razahi, Raman Kumar, Jasgurpreet Singh Chohan, Aditya Tiwary, Shubham Sharma, Changhe Li, R. A. Ilyas, M. R. M. Asyraf, S. Z. S. Zakaria

**Affiliations:** 1 Department of Civil Engineering, Chandigarh University, Mohali 140413, India; drsandeep1786@gmail.com (S.S.); iqbal.khairandish@gmail.com (M.I.K.); mustafa.musleh.r@gmail.com (M.M.R.); aditya.civil@cumail.in (A.T.); 2 University Centre of Research and Development, Department of Mechanical Engineering, Chandigarh University, Mohali 140413, India; ramankakkar@gmail.com (R.K.); jaskhera@gmail.com (J.S.C.); 3 Department of Mechanical Engineering, IK Gujral Punjab Technical University, Main Campus, Kapurthala 144603, India; 4 School of Mechanical and Automotive Engineering, Qingdao University of Technology, Qingdao 266520, China; sy_lichanghe@163.com; 5 School of Chemical and Energy Engineering, Faculty of Engineering, Universiti Teknologi Malaysia, Johor Bahru 81310, Malaysia; ahmadilyas@utm.my; 6 Centre for Advanced Composite Materials, Universiti Teknologi Malaysia, Johor Bahru 81310, Malaysia; 7 Institute of Energy Infrastructure, Universiti Tenaga Nasional, Jalan IKRAM-UNITEN, Kajang 43000, Malaysia; asyrafriz96@gmail.com; 8 Research Centre for Environment, Economic and Social Sustainability (KASES), Institute for Environment and Development (LESTARI), Universiti Kebangsaan Malaysia (UKM), Bangi 43600, Malaysia

**Keywords:** sisal, coir, rice straw fibers, waste marble, SEM topography, EDS analysis, marshall stability properties, drain down test, moisture sensitivity test

## Abstract

The present study investigates the preference index of natural fibers such as sisal, coir, and rice straw fibers in stone matrix asphalt mixtures (SMA), using waste marble as filler. Waste marble was used as the filler in asphalt mixtures and was crushed by abrasion machine and sieved according to SMA filler requirements. The SEM topography and EDS analysis of sisal, coir, and rice straw fibers were also carried out. The Marshall test was conducted, which is the most acceptable, cost-effective, and widely adopted method to estimate the optimum bitumen and to examine several Marshall Measures, such as flow value, voids filled with bitumen (VFB), stability, voids in mineral aggregate (VMA), and air voids (VA). Furthermore, tests were performed on the specimen with the optimum amount of bitumen, different percentages of fibers, and waste marble as filler to calculate drain down, moister sensitivity, and Marshall Stability. Multi-criteria decision-making (MCDM) techniques were implemented to obtain subjective and objective weights, which were further used to compute the values of the preference index of natural fiber contents. The outcomes revealed favorable results for the usage of marble dust as filler in Stone Matrix Asphalt (SMA). In addition, the preference index upshots are inclined toward the usage of rice straw over coir followed by sisal fiber. It was observed that the value of the preference index in rice straw at 0.3 varied from 0.918, 0.925, and 0.931 in rice straw using equal, objective, and subjective weights, respectively. The maximum drain down value observed is 0.335 based on ASTM-D 6390 and IRC-SP-79 are against 0.3 percent natural fiber. Moreover, as per the prescribed limit of MoRTH, because of the thin film around aggregates, moisture susceptibility characteristics, i.e., better resistance to moisture, were enhanced by more than 80%.

## 1. Introduction

Natural fibers are diverse and plentiful, as they can be obtained from a wide range of plants, trees, crops, and waste products [1]. Natural fibers have a (relatively) low density and cost, minimal energy consumption, and are biodegradable [2,3]. As a result, natural fibers have several technological advantages for use in composite materials. Incorporating natural fibers into cementitious composites further slows the release of carbon-rich elements into the atmosphere by storing their stored energy [4,5].

The most extensively used material in road construction and maintenance industry is Hot Mix Asphalt (HMA). Depending upon the aggregate gradation, the mix is divided into three major categories, namely the well-graded mix, gap-graded mix, and open-graded mix [6,7]. In the 1960s, Stone Matrix Asphalt (SMA) was developed in Germany, which is capable of preventing and resisting the wear and cracks in bituminous pavement caused by vehicle wheel load. It is composed of 70–80% coarse aggregates, 20–25% fine aggregates, 9–13% filler, 6–7% bitumen content and 0.3–0.5% stabilizing additives [8]. Various national and international investigations also showed that the SMA is more rutting-resistant and durable than dense-graded mixtures. It encouraged other countries, especially European countries, to use this unique mixture. Moreover, in the USA, in 1960, some transportation agencies made an investigative journey to Europe, and the performance of SMA was evaluated [9]. This led to detailed experimental and field investigations on SMA, and its excellent performance makes it one of the first to be selected for pavement designers. The high capacity and resistance to deformation, shear strength, and effective wheel load distribution in heavy traffic conditions in SMA mixtures are due to the high percentage of coarse aggregate availability, which results in more stone-to-stone contact and binder content than in dense-graded bituminous mixtures [10,11]. The higher binder content, thicker bitumen film, and lower air void content improve durability and flexibility. The addition of a small quantity of cellulose or mineral fibers prevents the drainage of bitumen during transport and placement [12]. The essential aspects, such as coarse aggregate skeleton, mastic composition, surface texture, and mixture stability, are related to the selection of aggregate gradation, filler and binder type, and proportion. SMA yields good rut resistance, durability, fatigue, and tensile strength, due to its coarse aggregate surface texture; it can be used in wet areas and locations where low-noise is expected [13,14,15]. Mineral fillers and additives have helped in the maximization of the binder by reducing binder drain down and improves durability as well.

In bituminous mixes, fillers and additives participate in two ways, based on their particle size; finer particles help enhance viscosity and consistency while coarser particles act as inert elements that fill voids and provide strength and impermeability [16]. Various researchers suggested that additives and fillers could demonstrate a different level of performance under pavement distresses based on their physical (gradation, porosity, specific gravity) and chemical (mineralogy) characterization [16,17,18,19]. Thus, a selection of the best additives and fillers and their proportions is vital, as it affects the cost (construction and maintenance) and performance of the pavement during its service life [20]. In the past two decades, various researchers have emphasized replacement of conventional materials and suggested industrial, commercial, agricultural, and domestic wastes as fully or partially replacement for the development of advanced materials. The most common waste materials suggested are coal fly ash [21], rice husk ash [22,23], brick dust [17,22], borogypsum [24], phosphogypsum [25], and green liquor dregs [18]. Although most of the waste (except phosphor-gypsum and green liquor dregs) showed promising performance in laboratory testing, it mostly depends on the origin or generation process, along with gradation and proportion of clay [26]. Intensive characterization of waste plays a vital role in defining its role as required [23].

In the present study, an attempt was made to gauge the performance of natural fibers (coir fiber, sisal fiber, and rice strew fiber) in a stone matrix asphalt mixture using waste marble as filler. The first phase of the study constitutes material characterization (via gradation analysis, physical properties of aggregates, and binder (VG30), along with SEM analysis for additives). Afterward, Marshall testing was performed to determine the optimum bitumen content (OBC), optimum fiber content (OFC), and volumetric analysis of mixes. The observed data were examined using multi-criteria decision-making (MCDM) methods by calculating subjective, objective, and equal weights of attributes. Thereafter, to evaluate the tensile strength and moisture susceptibility, an indirect tensile strength test (ITS) and tensile strength ratio (TSR) test were conducted. Subsequently, to gauge the drain down of the binder for loose mixture, a drain down test (as per MoRTH Specification) was performed.

## 2. Material and Experimentation

**Figure 1:**
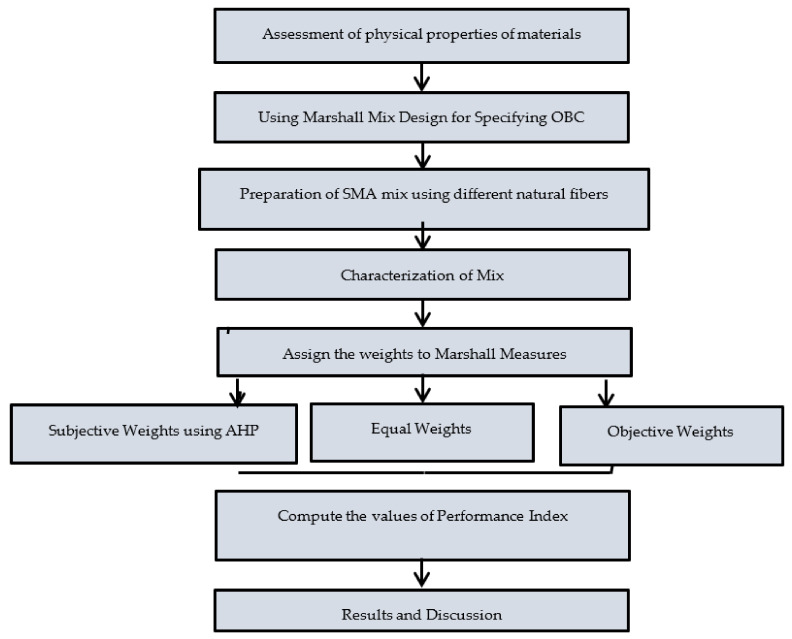
Work flow diagram.

The design process was based on the Marshall Mix design for specifying OBC and OFC of SMA mixtures, on the basis of Marshall Parameters as max stability, max flow, and specified range of air voids (MoRTH (5th revision)). As per the institute of asphalt manual (MS-2) and the Ministry of Road Transport and Highways (MoRTH) for SMA mixes, to reduce rutting influence and to improve mix performance, the considered range for air void is four percent [26,27]. The strategy flow diagram of the present study is shown in Figure 1.

### 2.1. Use of Aggregates

**Figure 2:**
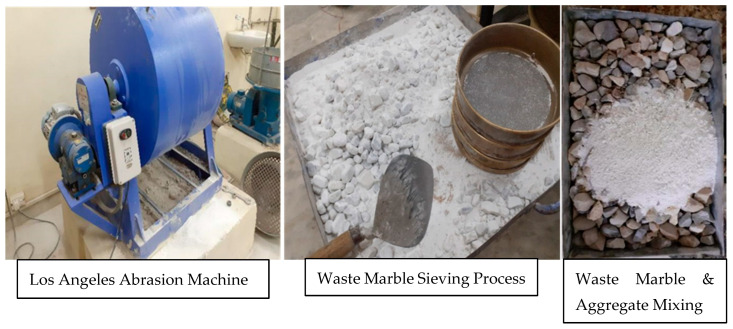
Mineral filler preparation.

Aggregate is the major component of bituminous mixes; 95 percent of SMA mixture is aggregate. In the preparation of SMA mixture, the first gradation of SMA mixture (the ingredients for SMA mixture i.e., Aggregates and Bitumen, were supplied by Singh Construction Materials Pvt. Ltd., Ludhiana, India) with a nominal maximum size (NMAS) of 19 mm was used, as per the guidelines of MoRTH (5th revision), which is presented in Table 1. Thereafter, aggregate that is to be used for road construction should withstand the physical requirements described by MoRTH; the limitations and results of the considered sample are incorporated in Table 2. Similarly, the fine aggregate considered has a bagged specific gravity of 2.48 and is procured in a laboratory by crushing stone in the Los Angeles abrasion test machine. Waste marble with a specific gravity of 2.63 as mineral filler is processed from construction waste by crushing in the Los Angeles abrasion test machine (Supplied by AIMCO Solutions Llp, Mohali, India, Model No.—AI714); details are shown in Figure 2. VG 30 grade bitumen (Supplied by Singh Construction Materials Pvt. Ltd., Ludhiana, India) is used in examination and results of specified physical properties (as described in MoRTH), for traffic loads of 20 to 50 MSA (according to IRC 37: 2018) and temperatures of 38–45 °C (as per IS 73); this is summarized in Table 3 [28,29].

Considering the importance of the additives in the SMA mixture, which has been proved by numerous studies, this study used three natural additives (sisal, coir, and rice straw fiber). To evaluate the presence of these additives with the presence of waste, marble dust (is powder waste, produced during the cutting of marble, so collected from Bhagwati Marble Store, Dhanas, Chandigarh) has been used as a filler in the mixture. Bituminous mixes’ overall performance is influenced by the mineral composition and microstructure of aggregate. The scanning electron microscope (SEM) is one of the most effective and extensively used electron microscopy technologies for obtaining images of a specimen by scanning the surface of the object with electron-focused beams. By interacting with atoms, SEM produces various signals that encompass related information about surface topography and chemical composition of the sample. To gather information regarding the chemical composition and microstructure morphology of aggregate samples, a JSM-IT500 SEM was used, which is a new model from JEOL’s TouchScope^TM^ Scanning Electron Microscope line, with resolution of 1.0 nm @ 15 kV and magnification of 12 to 1,000,000 times. The properties, characteristics, and effects of each are discussed below:

#### 2.1.1. Sisal Fiber

**Figure 3:**
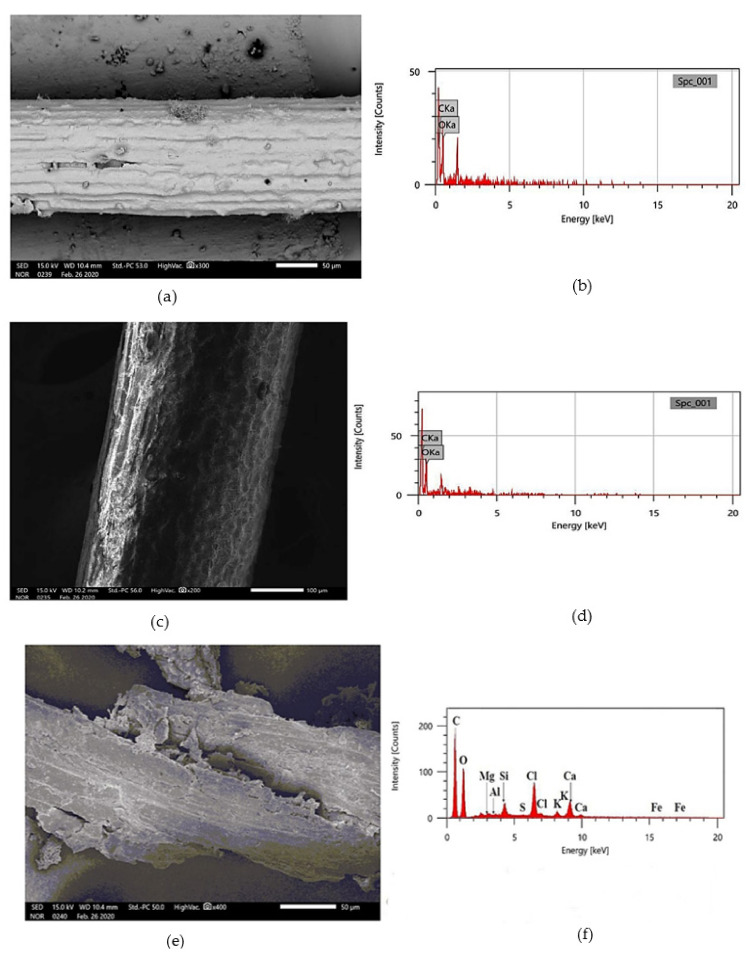
Sisal fiber: (**a**) SEM topography and (**b**) EDS analysis; brown coir fiber: (**c**) SEM topography and (**d**) EDS analysis; and rice straw fiber: (**e**) SEM topography and (**f**) EDS analysis.

Sisal fiber is a champion among the most comprehensively used common fibers. It is processed from the sisal plant; it is cellulose and yellowish. For an examination of the influence on engineering properties, a locally available modifier has been used. Figure 3a,b shows the result of the SEM and EDS analysis (feature of JEOL’s TouchScope^TM^ line) of sisal fiber. Figure 3a shows the magnified images of fiber at 300 times magnification at 50 μm scale. Through the EDS analysis of samples, which is available in Figure 3b, we show the chief amount of Cka and Oka.

#### 2.1.2. Coir Fiber

After soaking the coconut for three months, the outer skin is pulled out, called coir fiber. Based on the color, the coir fibers are bifurcated into two categories (white and brown fiber). Brown coir consists of more essence of lignin and is obtained from mature coconuts. As per the previous examination, it has been shown that coir fibers are thick, strong, and have high abrasion resistance. Figure 3c,d shows the result of the SEM and EDS analysis of coir fiber. Figure 3c shows the magnified images of fiber at 200 times magnification at a 100 μm scale. Through the EDS analysis of samples, which is available in Figure 3d, we show the chief amount of Cka and Oka.

#### 2.1.3. Rice Straw Fiber 

Rice straw, which is a by-product of rice production, is another natural and sustainable resource, each kilogram of milled rice produces 0.7–1.4 kg (Approximately) of rice straw depending on varieties, the stubble’s cutting height, and moisture content in the course of the harvest. It is generally used for concrete as reinforcement, but in this study, it will be used in an SMA mixture with a bulk density of 50 kg/m^3^. Figure 3e,f shows the result of the SEM and EDS analysis of rice straw fiber. Figure 3e shows the magnified images of fiber at 400 times magnification at a 500 μm scale. Through the EDS analysis of samples, which is available in Figure 3f, we show the chief amount of C, O along with Chloride (Cl), Calcium (Ca), Silica (Si), Magnesium (Mg), Aluminum (Al), Potassium (K) followed by a little essence of Iron (Fe).

## 3. Volumetric Analysis and Marshall Characterizations 

The Marshall Mix design standard ASTM-D 1559 (via Marshall Testing Machine (AIM 550-6), manufactured by AIMIL Instrumentation and Technology) is used for the preparation of SMA specimens [30]. As per the specification of MoRTH, selected gradation (such as coarse aggregate, fine aggregate, and waste marble dust as filler in appropriate quantities) for stone matrix asphalt (SMA) (presented in Table 2), is heated in the range of 154–160 °C. 

The heated aggregate is then mixed with different bitumen percentages according to the total weight of granular material (such as 1200 g), each bitumen percentage is mixed with three specimens. The asphalt mixture was poured into a special Marshall Test cylindrical mold; the compaction process is accomplished by applying 50 blows to either side of specimens, and after 24 hr. dimensions and weights are evaluated to gauge the volumetric properties. This was followed by the Marshall Test, which was performed as per ASTM-D 6927 guidelines to gauge the stability and flow behavior of each sample [31]. Mixtures with all binder contents were made according to the SMA mixture requirements and hence only air void-based MoRTH and asphalt institute manual (MS-2) were followed as the critical factors for optimum bitumen content (OBC), and bitumen content corresponding to 4% air voids is taken as the OBC (MS-2, 2014). The volumetric and Marshall properties are shown in Table 4.

To test the behavior of fibers (sisal, coir, rice straw) in marble as small filler mixtures and considering the obtained optimum bitumen content (OBC), sisal, coir, and rice straw fibers are covered with slow setting emulsion (SS-1) and afterward placed in a hot air oven (procured from Anadigi Solutions Private Limited, Chandigarh, India) for 24 h at 110 °C. Thereafter, the fibers are cut to the length of 8 mm and used in the mixture base in varying percentages, the optimum fiber content according to previous studies was reported to be less than 0.3% by weight of mixture with conventional fillers. However, in this study, marble waste was used as filler, and fibers were cut manually into small pieces (8 mm in length) to ensure complete blending with aggregate and bitumen during the mixing process. This particular procedure was adopted based on the experience of a series of initial trial mixes. Furthermore, the fiber content in the mix was combined with different concentration ratios from 0.25% to 0.4% with an interval of 0.5% (i.e., 0.25%, 0.3%, 035%, and 0.4%) by weight of the mixes. Emulsion coating was adopted, based on the organic nature of the material. Figure 1 presents the original and coated state of the fibers. The process mentioned above is repeated with the different options, such as fiber availability and bitumen content, to determine the volumetric and Marshall Properties of the fibers in different percentages and the marble as a filler corresponding to OBC is presented in Table 5.

## 4. Indirect Tensile Strength (ITS) and Indirect Tensile Strength Ratio (TSR)

The Indirect tensile strength (ITS) evaluates the tensile strength and potential for rutting of Marshall Specimens under compressive loading on the vertical diametrical plane at a specific load rate (50 ± 5 mm/min) and temperature (25 °C); indirect tensile strength is standardized in ASTM-D 6931 specification [32]. As per AASHTO-T 283 and ASTM-D 4867/D4867M guidelines, specimens are conditioned (60 °C for 24 h) to evaluate the moisture susceptibility characteristics (ASTM-D 4867/D-4867M and AASHTO-T 283). The ratio of ITS results in conditioned and unconditioned specimens, shown as the indirect tensile strength ratio (TSR), which is a measure of moisture strength of SMA mixtures. From the test results (refer to Table 6) of three natural fibers with waste marble as a filler along with optimum bitumen content (OBC), it can be concluded that the fibers with optimum fiber content (OFC) showed the best performance, [33,34].

## 5. Drain Down

To gauge the extent of drain down of the binder in a loose mixture, a drain down test was recommended by MoRTH (5th revision). The investigation was conducted according to ASTM-D 6390 in a standard wire basket at 160–170 °C, [35]. Approximately 1200g loose SMA Mix sample was prepared and dispensed in the basket. Thereafter, the basket with the sample is placed over an oven plate kept at a prescribed temperature; after one hour, the material is collected in the catch plate. Afterward, the final weight, i.e., drained down, the value of mixtures was calculated by dividing the initial and final weight. The drain down test results of the present study is reported in Table 7.

## 6. Implementation of Multi-Criteria Decision-Making Method

The use of MCDM methods is a common practice to attain the best alternatives based on available attributes. The literature supports various methods to compute the subjective and objective priority of the attributes. In the present work, subjective weights, objectives weights, and equal weights are considered to evaluate the optimum value of fibers contents in Natural fiber. The preference index of alternates is computed using different weights and results are compared.

### 6.1. Implementation of AHP

The Analytic Hierarchy Process (AHP) is an advanced and structured technique of decision-making presented by Thomas Saaty in the late 1980s [36]. AHP attracted the interest of researchers primarily due to its simplicity and effectiveness. AHP can resolve complicated decision problems in the manufacturing environment. The output of AHP is validated by consistency analysis. 

Step 1. Development of pairwise matrix

The pairwise matrix was developed using Satty’s nine-point scales. The pairwise comparisons are given in terms to present the importance of attributes with each other. In this work, 10 comparisons were established between five attributes, as shown in Table 8.

Step 2. Subjective weighs and measurement of consistency 

The consistency ratio as shown in Table 9 is calculated for the validation process of the pairwise comparison matrix. It was suggested to find the value of the maximum eigen vector λ_max_ and consistency index (CI).

The formula used for the calculation of CI is given as [37,38,39,40,41,42,43,44,45]:

CI = (λ_max_ − n)/(n − 1)

The ratio of consistency index and random consistency index gives the value of the consistency ratio.

### 6.2. Computation of Objective Weights

Rao and Patel (2010) presented an advanced technique to find the objective weights of each attribute using a statistical variance [37]. This technique was proposed to avoid errors in subjective weights. The guidelines were presented to find a preference index using objective and subjective weights as well. The findings of the aforementioned methodology were used for comparison with existing results to validate the performance and accuracy of the proposed work. Various examples were demonstrated to validate the proposed work. The step-wise computational work was described to show the simplicity of finding preference index values of the attribute.

Step 1 Normalized Matrix (*N_ij_*)

The data present in the decision table may have different units; therefore, normalizing the data is an essential step in a decision-making problem. The normalized matrix is developed using Equation (1) and the values are shown in Table 10.

(1) $$N_{ij}=(D_{ij}/\sum_{i=1}^{n}D_{ij})$$

Step 2 Calculate the statistical variance and objective weight

Statistical variance implies the distribution of data around the average values when calculated using standard statistical tools. This can be calculated by taking an average of all the sums of squared deviations from the mean value. The major and significant difference between variance and range is that the latter only considers the extreme value, while the former analyzes all the data points and afterward decides the distribution. The variance and objective weights can be computed using Equations (2) and (3) and the values are shown in Table 11.

(2) $$V_{i}=\frac{1}{n}{\sum_{i=1}^{n}\left\{N_{ij}-\left(N_{ij}\right)mean\right\}}^{2}$$

(3) $$W_{i}=\frac{V_{i}}{\sum_{i=1}^{n}V_{i}}$$

Step 3 Preference Index value

The weights are assigned to each attribute and the preference index of attributes as shown in Table 12 is calculated using the weighted sum concept. The preference index helps to rank alternates. The preference index (*P_i_*) is computed using the following equations. Equations (4)–(6) are used to find preference index using subjective, objective, and equal weights, respectively [41,42,43,44,45,46,47,48,49,50,51,52,53].

(4) $$P_{i}^{o}=\sum_{j=1}^{m}W_{j}^{o}D_{ij}^{**}$$

(5) $$P_{i}^{s}=\sum_{j=1}^{m}W_{j}^{s}D_{ij}^{**}$$

(6) $$P_{i}^{e}=\sum_{j=1}^{m}W_{j}^{e}D_{ij}^{**}$$

where $D_{ij}^{**}=\left[\frac{D_{ij}^{*b}}{\left(D_{ij}^{*b}\right)\max}\right]$ for beneficial attributes only & $D_{ij}^{**}=\left[\frac{\left(D_{ij}^{*nb}\right)\min}{D_{ij}^{*nb}}\right]$ for non-beneficial attributes only. $D_{ij}^{*b}$ presents the maximum value of the *j*th beneficial attribute and $D_{ij}^{*b}$ present the minimum value of the *j*th beneficial attribute.

## 7. Results and Discussions

### 7.1. Marshall Properties of Bituminous Mixes at Optimum Bitumen Contents

The lab mix design is investigated using stability and flow parameters, as well as air void (VA) in the overall mix and void in mineral aggregate (VMA). However, in practice, stability and flow performance are prioritized in order to comprehend Marshall Mix’s recital. The load carrying capability of a mix is defined by its stability value, hence adequate mix stability is a requirement for a road since it aids in resistance to cracking, rutting, and bleeding [37,38,39,40,41,42,43,44,45,46,47,48]. The volumetric analysis and Marshall features of SMA are shown in Table 4. The Optimal Bitumen Percentage (OBC) is 5.95 percent, according to the analysis based on the Standard design technique of road mix design (ASTM-D 6927 standards) or measurement of volumetric parameters, marshal stability, and flow value by including waste marble as filler.

It is a well-known fact that fibers are incorporated in SMA mixtures as reinforcement, to enhance durability and deformation resistance under load. However, the optimal dosage is vital; otherwise, results could be fatal or less efficient (less resistance under loading even lower than the conventional mixes). The results of the Marshall volumetric investigation at Optimum Bitumen Content (OBC) for all three different fibers (i.e., sisal, coir, and Rice Straw) are shown in Table 5. In the case of Sisal fiber, the Marshall stability was observed at a maximum i.e., 8.766 kN against 0.40 percentage of sisal fiber, whereas in the case of coir fiber, the Marshall stability value (10.964kN) was maximum at 0.25 percentage of coir fiber. However, as per MoRTH, 0.3% is the minimum fiber content by weight of the total mix. Therefore, 10.710 kN is the maximum valid value of Stability achieved against 0.3 percent of Coir Fiber. Moreover, in the case of Rice straw, the maximum value of stability, i.e., 10.70 kN, was achieved against 0.3 percentage of coir straw fiber.

Similarly, the flow values were obtained well within the prescribed limits (ASTM-D 6927). Except in the case of SMA Mix, prepared with 0.25 percent of Rice Straw Fiber, as the flow volume marginally exceeds the limit (i.e., 4.010 mm). However, as mentioned previously, MoRTH has the recommended minimum weightage of fiber, which should be 0.3 percent, so the mix with 0.25 percentage of Rice straw fiber could be eliminated.

**Figure 4:**
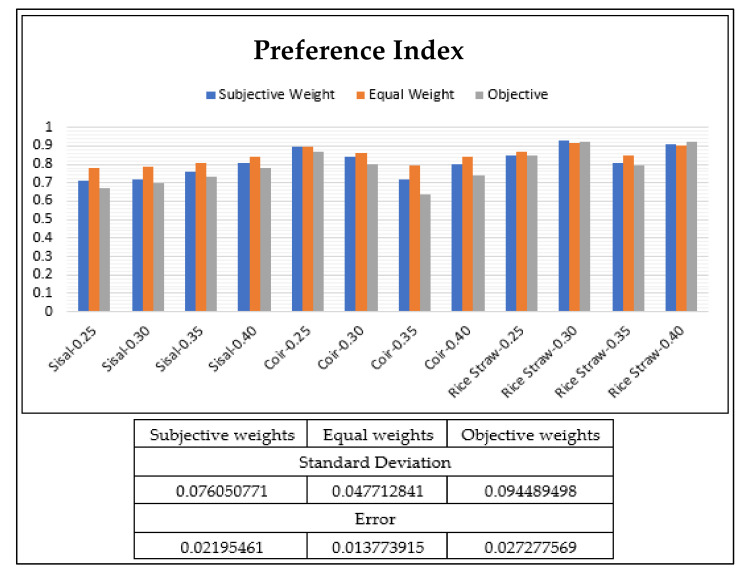
Preference Index values.

The comparative chart preference index is shown in Figure 4. The preference index in the case of sisal fiber (with 0.40 percent dosage) is obtained as 0.808, 0.839, and 0.782 against equal, objective, and subjective weights, respectively. Whereas in the case of coir fiber (at 0.30 percent dosage), the preference index was measured as 0.844, 0.863, and 0.798 against equal, objective, and subjective weights, respectively. Although the values were higher for 0.25 percent of Coir Fiber, but as discussed previously, according to MoRTH guidelines the same has been neglected. It was observed that the value of the preference index in rice straw-0.3 is varied from 0.918 to 0.931 using different weights. The preference index is 0.918, 0.925, and 0.931 in rice straw using equal, objective, and subjective weights, respectively. Therefore, eventually comparing all three fibers, the preference index results favor Rice Straw Fiber reinforcement

### 7.2. Drain down Test

In the laboratory investigation of drain down, the reaction of SMA mixtures with various fibers and waste marble as mineral filler showed that the drain down characteristics of mixtures decreases by increasing the fiber content [38,39,40,41,42,43,44,45,46,47,48,49,50,51,52,53,54]. The maximum drain down value (0.335) based on ASTM-D 6390 and IRC-SP-79 are against 0.3 percent of natural fiber. Therefore, it was determined that by adding the optimal fiber content (OFC) to the mixtures, the drain down properties will improve. The test results of all mixtures are shown in Table 7 and also show that with the increase of fiber percentage the drain down value decrease. It can be seen that for a zero fiber percentage mix, the average value for three samples recorded was 1.66, whereas, for fiber-mixed samples, the value of drain down test decrease considerable for all the three fibers i.e., sisal, coir, and rice straw, specifically at 0.4 percent fiber addition, the drain down test result recorded nil value.

### 7.3. Indirect Tensile Strength and Tensile Strength Ratio

The laboratory evaluation of indirect tensile strength (ITS) of the SMA mixtures provides opportunities to measure the tensile strength of SMA mixtures in the unconditioned state (Normal Specimens) [53,54,55,56,57,58,59]. Moreover, the specimens are tested in conditioned (water bath-maintained specimens), the ratio of ITS values is shown as tensile strength ratio (TSR), and which is the amount of moisture resistance of SMA mixtures. The result is presented in Table 6, where it is shown that better resistance to tensile strength and moisture is achieved by SMA mixes cast with optimum fiber content (OFC). As per the prescribed limit of MoRTH, moisture susceptibility characteristics (better resistance to moisture) should be greater than 80%. As in Table 6, for both unconditioned and conditioned samples the best results were observed at 0.3 percent fiber addition. The values 1.2, 1.3, and 1.33 were recorded for unconditioned samples and 1.13, 1.20, and 1.23 for conditioned samples of sisal, coir, and rice straw fiber, respectively. Therefore, they have registered a hick of 20, 30, and 33 percent for unconditioned and 25, 28, and 30 percent for conditioned samples prepared using sisal, coir, and rice straw fiber, respectively. Similarly, at 0.3 percent fiber content, the SMA Mixes showed a maximum resistance of moisture susceptibility for all the three fibers, i.e., 94.02, 93.45 and 91.74 percent for sisal, coir and rice straw, respectively. In addition, the resistance of moisture susceptibility increased by 9, 6, and 7 percent for sisal, coir, and rice straw, respectively compared to zero percent of fiber. Thus, Natural fibers have several technological advantages for use in composite materials. Incorporating natural fibers into cementitious composites further slows the release of carbon-rich elements into the atmosphere by storing their stored energy and enhances the strength and durability for several applications [58,59,60,61,62,63,64,65,66,67].

## 8. Conclusions

The examination showed the scope for effective usage of sisal, coir, and rice straw fibers, along with marble waste in SMA mixes. Marble waste as a mineral filler showed acceptable performance in SMA mixtures and is recommended for the construction of SMA roads. The present investigation draws the following conclusions:

The optimal bitumen content, shown in Table 4. is 5.95 percent. The key considerations for the decision were Air Void (VA), which should not exceed 4 percent, minimum flow value (3.666), along with maximum stability (i.e., 8.688).

The addition of the fiber also showed a considerable impact on the performance of the SMA Mix. The flow value has considerably reduced and the stability value increased with the addition of all three natural fibers. Table 5 shows that with the addition of sisal fiber (at 0.4 percent) the stability value has increased by almost 10 percent. Similarly, the stability value increased by 50 percent for Coir Fiber and Rice Straw at 0.35 and 0.3 percent dosage, respectively. Table 5 also revealed that the Flow Value for SMA mixes prepared via incorporating fibers reduced considerably and results are well within the prescribed limits of MoRTH. However, in a comparative analysis of all three natural fibers used via consideration of Marshall Mix design and test criteria, it was observed that adding 0.3 fibers of rice straw to SMA mixtures significantly improve the properties of Marshall.

Along with the Marshall Characterization, ITS and TSR of modified SMA mixtures also recorded the best performance at optimum fiber content (OFC) in the conditioned and unconditioned samples. As shown in Table 6 for Unconditioned Samples, the ITS increased by 8, 10 and 12 percent for sisal, coir, and rice straw, respectively. Consequently, ITS also increased by 7–8 percent for all three fibers, in the conditioned scenario. In line with the ITS results, the TSR has increased by 9–10 percent for modified SMA mixes.

Similarly, the drain down test result of SMA mixtures registered a reduction in value by increasing the amount of fiber; as fiber causes drain interruption in the mixture. Table 7 shows that the addition of 0.3 percent of fiber has reduced the drain value by 65, 90, and 52 percent for sisal, coir, and rice straw fiber, respectively.

The value of the preference index is higher in rice straw at −0.3 and followed by rice straw at −0.40. The values of sisal 0.25 have the lowest preference index in all three conditions i.e., using equal, subjective, and objective weights.

## Figures and Tables

**Table 1:** Aggregate Gradation as per MoRTH (5th revision).

IS Sieve Size (mm)	Cumulative % by Weight of Total Aggregate Passing	
	Range	Adopted
26.5	100–100	100
19	90–100	95
13.2	45–70	57.5
9.5	25–60	42.5
4.75	20–28	24
2.36	16–24	20
1.18	13–21	17
0.6	12–18	15
0.3	10–20	15
0.075	8–12	10

**Table 2:** Physical Properties of Aggregate.

Property	Test Value	MoRTH Limits	Test Method
Aggregate impact value (%)	14.2		IS:2386 (Part 4)—1963
Aggregate crushing value (%)	12.8		IS:2386 (Part 5)—1963
Los Angles abrasion value (%)	18.5		IS:2386 (Part 4)—1963
Flakiness index (%)	18.6		IS:2386 (Part 1)—1963
Elongation index (%)	22		IS:2386 (Part 1)—1963
Water absorption (%)	1.33		IS:2386 (Part 3)—1963
Specific gravity	2.6		IS:2386 (Part 3)—1963

**Table 3:** Properties of VG30 Bitumen.

Property	Test Value	MoRTH Limits	Test Method
Penetration at 25 °C (0.1 mm)	68		IS:1203—1978
Specific gravity	1.03		IS:1202—1978
Softening point (°C)	49		IS:1205—1978
Ductility			IS:1208—1978

**Table 4:** Volumetric analysis and Marshall Characteristics of SMA.

Property	Bitumen Content by Weight of Aggregate			
	5	5.5	6	6.6
VA (%)	4.969	3.946	4.982	5.593
VMA (%)	17.052	16.429	17.703	18.569
VFB (%)	70.855	75.979	71.854	69.876
Stability (KN)	7.595	8.688	8.382	7.710
Flow (mm)	4.963	3.770	3.666	5.1433
OBC (%)	5.95			

**Table 5:** Volumetric analysis and Marshall Characteristic of Fibers at OBC.

Natural Fibers Type	VA (%)	VMA (%)	VFB (%)	Stability (kN)	Flow (mm)
Sisal-0.25	3.835	15.982	76.001	7.533	2.500
Sisal-0.30	4.041	15.850	74.500	7.164	3.000
Sisal-0.35	4.535	16.076	71.787	7.900	3.500
Sisal-0.40	4.859	16.223	70.045	8.766	4.000
Coir-0.25	3.519	15.818	77.740	10.464	3.696
Coir-0.30	3.937	16.000	75.393	10.610	3.263
Coir-0.35	5.876	17.459	66.342	8.966	2.726
Coir-0.40	5.654	17.025	66.789	10.033	3.546
Rice Straw-0.25	5.072	17.097	70.334	7.883	4.030
Rice Straw-0.30	3.980	16.04	75.182	10.700	3.680
Rice Straw-0.35	5.615	17.299	67.536	7.833	3.580
Rice Straw-0.40	3.964	15.574	74.543	9.633	3.880

Note: 0.3% is the minimum fiber content by weight of total mix, according to MoRTH.

**Table 6:** Indirect tensile strength (ITS) test result.

Fiber Percent	ITS Unconditioned			ITS Conditioned			TSR		
	Natural Fibers			Natural Fibers					
	Sisal	Coir	Rice Straw	Sisal	Coir	Rice Straw	Sisal	Coir	Rice Straw
0	1.01	1.01	1.01	0.887	0.887	0.887	87.79	87.79	87.79
0.25	1.0149	1.183	1.1829	0.9253	1.0691	1.075	91.168	90.374	90.903
0.3	1.202	1.292	1.339	1.13	1.207	1.2284	94.02	93.45	91.739
0.35	1.1566	1.147	1.439	1.059	1.0462	1.339	91.579	91.211	93.01
0.4	1.0877	1.0869	1.251	0.986	0.981	1.12	90.691	90.241	89.556

**Table 7:** Drain down test result.

Fiber Content	Natural Fiber Type		
	Sisal	Coir	Rice Straw
0	1.658	1.658	1.658
0.25	0.251	0.176	0.2187
0.3	0.025	0.0167	0.0335
0.35	0.008	0.0084	0.0167
0.4	0.00	0.00	0.00

**Table 8:** Pairwise comparison matrix.

	VA	VMB	VFB	ST	FW
VA	1.000	4.000	3.000	0.330	0.330
VMB	0.250	1.000	2.000	0.200	0.200
VFB	0.333	0.500	1.000	0.250	0.250
ST	3.030	5.000	4.000	1.000	1.000
FW	3.030	5.000	4.000	1.000	1.000

**Table 9:** Subjective weights and consistency analysis.

	Subjective Weights	Consistency Results
VA	0.194	Total comparisons = 10 CR = 8.3% λmax = 5.373
VMB	0.112	
VFB	0.064	
ST	0.315	
FW	0.315	

**Table 10:** Normalized matrix.

	VA	VMB	VFB	ST	FW
Sisal-0.25	0.070	0.081	0.088	0.070	0.055
Sisal-0.30	0.074	0.081	0.086	0.066	0.066
Sisal-0.35	0.083	0.082	0.083	0.073	0.077
Sisal-0.40	0.089	0.083	0.081	0.081	0.088
Coir-0.25	0.064	0.081	0.090	0.101	0.081
Coir-0.30	0.072	0.081	0.087	0.099	0.072
Coir-0.35	0.107	0.089	0.077	0.083	0.060
Coir-0.40	0.103	0.087	0.077	0.093	0.078
Rice Straw-0.25	0.092	0.087	0.081	0.073	0.111
Rice Straw-0.30	0.073	0.082	0.087	0.099	0.103
Rice Straw-0.35	0.102	0.088	0.078	0.072	0.101
Rice Straw-0.40	0.072	0.079	0.086	0.089	0.107

**Table 11:** Values of variance and objective weights.

	Statistical Variance	Objective Weights
VA	0.000203313	0.289
VMB	1.01919 × 10^−05^	0.014
VFB	1.87314 × 10^−05^	0.027
ST	0.000146496	0.208
FW	0.000324883	0.462

**Table 12:** Preference index of alternates.

Natural Fibers Type	Subjective Weights	Equal Weights	Objective Weights
Sisal-0.25	0.709	0.778	0.674
Sisal-0.30	0.721	0.784	0.700
Sisal-0.35	0.759	0.807	0.733
Sisal-0.40	0.808	0.839	0.782
Coir-0.25	0.897	0.899	0.872
Coir-0.30	0.844	0.863	0.798
Coir-0.35	0.720	0.792	0.635
Coir-0.40	0.804	0.842	0.736
Rice Straw-0.25	0.846	0.867	0.851
Rice Straw-0.30	0.931	0.918	0.925
Rice Straw-0.35	0.807	0.845	0.791
Rice Straw-0.40	0.911	0.904	0.924

## Data Availability

No data were used to support this study.
